# Post-radiotherapy suggests a possible difficult airway even with an asymptomatic supraglottic change

**DOI:** 10.1186/s40981-022-00592-7

**Published:** 2022-12-29

**Authors:** Ayumi Oishi, Yukio Nomoto, Chiaki Nemoto, Satoki Inoue

**Affiliations:** 1 The Junior Resident Center, Ohara General Hospital, 6-1 Ohomachi, Fukushima, 960-8611 Japan; 2 Department of Otorhinolaryngology, Head and Neck, Ohara General Hospital, 6-1 Ohomachi, Fukushima, 960-8611 Japan; 3 Department of Anesthesiology, Ohara General Hospital, 6-1 Ohomachi, Fukushima, 960-8611 Japan; 4 Department of Anesthesiology, Fukushima Medical University, 1 Hikarigaoka, Fukushima, Fukushima 960-1295 Japan

**Keywords:** Airway management, Post-radiotherapy, Supraglottic change

## Introduction

Airway changes due to radiotherapy, even after several years, take various forms. One of the difficulties we encounter is the airway management [1, 2]. Most of these cases involve laryngeal edema, fibrotic changes, and restriction of neck flexion. With these physical findings, we could expect a difficult airway (DA) before anesthesia induction. Here, we describe an asymptomatic, peculiar supraglottic change due to previous radiotherapy.

## Case presentation

**Fig. 1:**
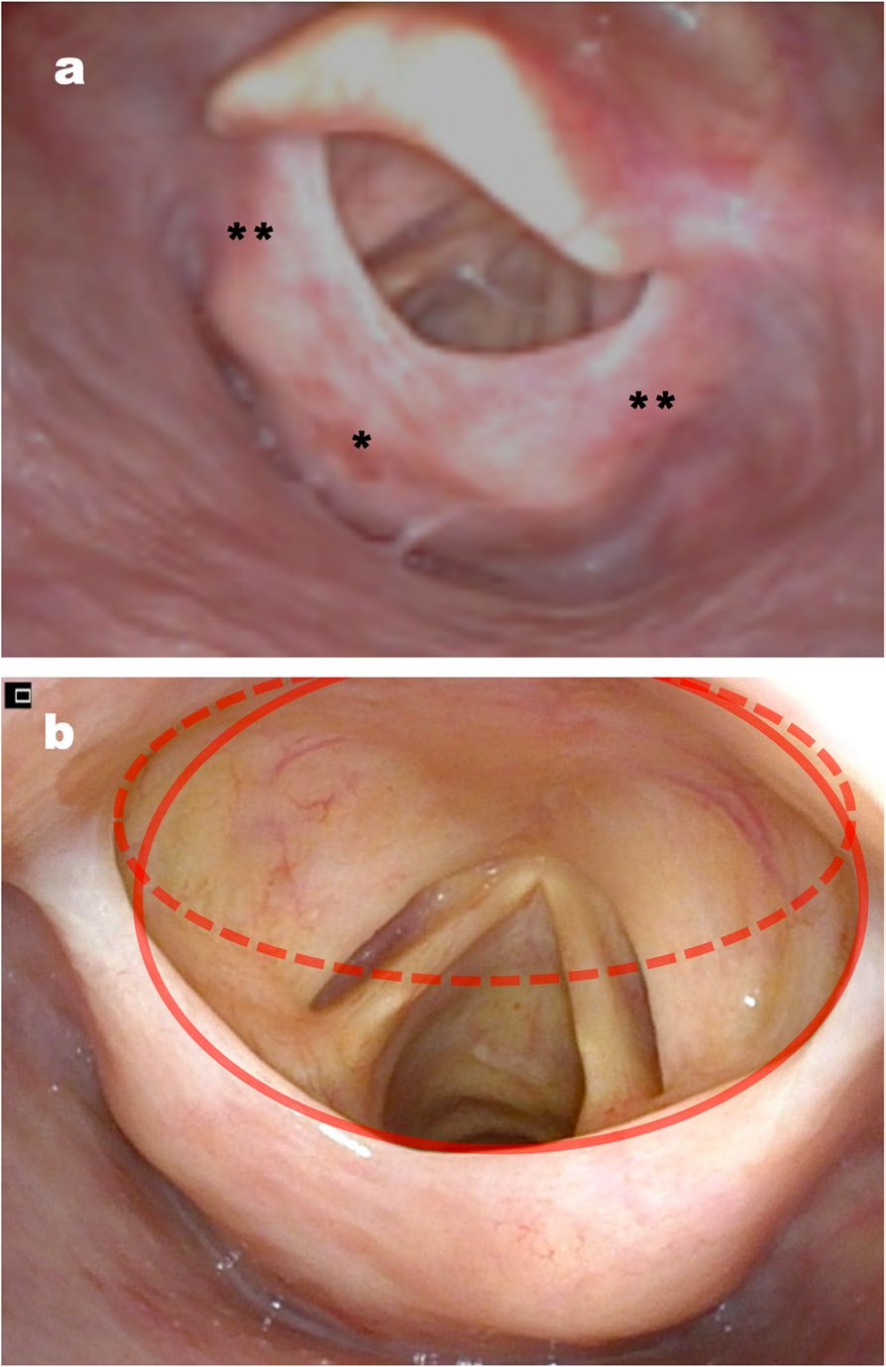
Preoperative flexible fiberscope image. **a** The wide angle of glottis view by preoperative flexible fiberscope. Radiotherapy for hypopharyngeal cancer elicited a cicatricial change in the arytenoid region^*^ to bilateral *aryepiglottic fold* ^**^ over a long period of time. Even cylindrical cicatricial change above vocal cord was observed, we could not evaluate this change at the physical examination 1 day before surgery. **b** The close angle of glottis view by preoperative flexible fiberscope. The change of shape in supraglottic hyperplasic cicatricial tissue during video laryngoscope exposer. As we inserted McGRATH® and tried to visualize the vocal cord, the red line changed to the red dotted line when we pulled the epiglottis upwards. Hyperplasic tissue was pulled up and narrowed the entrance to the trachea

An 83-year-old woman (height = 155 cm; bodyweight = 55 kg) had undergone radiotherapy (66 Gy) for hypopharyngeal cancer 16 years previously. Cancer recurrence was found, and tumor resection using direct laryngoscopy was scheduled. Physical examination 1 day before surgery did not reveal findings that suggested a DA, only previous radiotherapy. Fibrotic changes in the neck, restriction of neck flexion, hoarseness, or difficulty in swallowing were not observed. The image of preoperative computerized tomography scan around the epiglottis did not indicate any abnormalities which we might suspect DA. However, preoperative flexible fiberoptic laryngoscopy showed a supraglottic change (Fig. 1a).

The airway was open, and we assessed the risk of difficulty in ventilation and intubation to be low. Anesthesia was induced with propofol and remifentanil, and then, rocuronium was administered. Mask ventilation did not pose problems. During intubation, to obtain a clear view of the vocal cords (VCs), the epiglottis was pulled upwards by a video laryngoscope (McGRATH®; Aircraft Medical, Edinburgh, UK). Supraglottic hyperplasic cicatricial tissue was also pulled up and interfered with the VC view, and a narrowed airway hampered intubation with an endotracheal tube (internal diameter (ID) = 6.0 mm) (Fig. 1b). We tried again without pulling up the epiglottis and then intubated with a microlaryngeal endotracheal tube (ID = 5.0 mm; Covidien, Dublin, Ireland). Intubation was completed with second attempts of laryngeal exposure, and desaturation was not observed during intubation. The operation was trouble-free, and she was discharged from hospital on postoperative day 6.

## Discussion

We did not find post-radiotherapy alterations (e.g., restriction of neck movement, fibrotic changes, voice change) upon physical examination 1 day before surgery. In most cases after radiotherapy, dysphagia due to supraglottic stenosis occurs [3, 4]. Before we undertook endoscopy, we did not expect a supraglottic change. One should consider encountering a DA in a patient who has undergone radiotherapy in the past. Preoperative endoscopy findings suggested there was sufficient space to pass an endotracheal tube through supraglottic hyperplasic tissue. However, laryngoscopy-based exposure of hyperplasic tissue in the VCs narrowed the entrance to the trachea. Thus, we tried again with less force to locate only the VCs, and intubation was undertaken with a bent, smaller tracheal tube with a stylet. Awake fiberoptic intubation, which does not require laryngoscopy-based exposure, might allow intubation of tracheal tube to a pre-determined size. Whether we could have completed tracheal intubation by conventional laryngoscopy (which requires clearer exposure) in this patient is not known.

## Conclusions

We described supraglottic hyperplasic changes after radiotherapy in an asymptomatic patient. The preoperative supraglottic view observed by a flexible fiberoptic endoscope may change when intubation using a laryngoscope is done.

## Data Availability

Not applicable.

## Abbreviations

DA: Difficult airway
VCs: Vocal cords
ID: Internal diameter
